# Consequences of quantum noise control for the relaxation resonance frequency and phase noise in heterogeneous Silicon/III–V lasers

**DOI:** 10.1038/s41598-021-03314-8

**Published:** 2022-01-10

**Authors:** Dongwan Kim, Mark Harfouche, Huolei Wang, Christos T. Santis, Yaakov Vilenchik, Naresh Satyan, George Rakuljic, Amnon Yariv

**Affiliations:** 1 Department of Applied Physics and Materials Science, California Institute of Technology, Pasadena, CA 91125 USA; 2 Department of Electrical Engineering, California Institute of Technology, Pasadena, CA 91125 USA; 3 Telaris Inc., Santa Monica, CA 90403 USA

**Keywords:** Lasers, LEDs and light sources, Optical physics

## Abstract

We have recently introduced a new semiconductor laser design which is based on an extreme, 99%, reduction of the laser mode absorption losses. In previous reports, we showed that this was achieved by a laser mode design which confines the great majority of the modal energy (> 99%) in a low-loss Silicon guiding layer rather than in highly-doped, thus lossy, III–V p${}^+$ and n${}^+$ layers, which is the case with traditional III–V lasers. The resulting reduced electron-field interaction was shown to lead to a commensurate reduction of the spontaneous emission rate by the excited conduction band electrons into the laser mode and thus to a reduction of the frequency noise spectral density of the laser field often characterized by the Schawlow–Townes linewidth. In this paper, we demonstrate theoretically and present experimental evidence of yet another major beneficial consequence of the new laser design: a near total elimination of the contribution of amplitude-phase coupling (the Henry $\alpha$ parameter) to the frequency noise at “high” frequencies. This is due to an order of magnitude lowering of the relaxation resonance frequency of the laser. Here, we show that the practical elimination of this coupling enables yet another order of magnitude reduction of the frequency noise at high frequencies, resulting in a quantum-limited frequency noise spectral density of 130 Hz$^2$/Hz (linewidth of 0.4 kHz) for frequencies beyond the relaxation resonance frequency 680 MHz. This development is of key importance in the development of semiconductor lasers with higher coherence, particularly in the context of integrated photonics with a small laser footprint without requiring any sort of external cavity.

## Introduction

The semiconductor laser (SCL) has become and, very likely, will continue to be, in the foreseeable future, the linchpin of optoelectronics^1–5^. A few major obstacles, however, remain before the promise of CMOS-like Photonic Integrated Circuits (PICs) can be realized. Chief among these problems is: low-coherence, the dependence on external isolators to reduce optical feedback, and the coherence-reducing amplitude-phase coupling. Recently^6,7^, we have shown how the “removal” by modal design of optical energy from the lossy III–V material to low-loss material, Silicon in our example, reduces the frequency noise due to spontaneous emission and improves the field coherence by some three orders of magnitude, with the improvement being limited by the residual losses of the Silicon (or, more precisely, by the Q-factor of the laser mode). An unexpected bonus of the high-Q laser was its improved, 20–25 dB in our laboratory-fabricated lasers, insensitivity to optical feedback^8,9^. This improvement is again limited by the achievable intrinsic Q of the laser mode.

In this paper, we provide theoretical arguments and experimental evidence that the reduced interaction of the excited electrons in the SCL with the quantum-mandated zero-point field of the laser mode in our laser design leads, additionally, to a practical elimination of the amplitude-phase coupling at frequencies above that the relaxation resonance^10,11^. The order, or orders, of magnitude improvements in these three key metrics of the SCL are archived while maintaining its small size and the CMOS-fabrication compatibility. Taken together, they pave the way to a new generation of SCLs with intrinsic ultra-high-Q values as the main enablers of high-coherence photonic integrated circuits^12^.

In this paper, we take an ab-initio look at the relationships between some of the key attributes of the semiconductor laser; specifically, the current modulation response, phase-amplitude coupling, relaxation resonance, and the frequency noise (or phase noise). In the experimental section, we describe how the theoretical results are applied to the design of semiconductor lasers and present the measured relevant characteristics of these lasers. We use the heterogeneous Silicon/III–V laser as a proof-of-concept platform.

## Semiconductor laser theory

### Relaxation resonance frequency and the Schawlow–Townes linewidth

A convenient starting point in the analysis of the phase noise of a semiconductor laser is to consider the problem of the current modulation response. We start with the set of coupled equations for the number of photons $n_{l}$ in the lasing mode of the laser resonator and for the number of inverted electrons $n_{{\text {e}}}$ in the active regions (Equation 15.5-1^13^),

1 $$\begin{aligned} \frac{dn_{{\text {e}}}}{dt}&= \eta _{{\text {i}}} \frac{I}{q} - \frac{n_e}{\tau _{{\text {e}}}} - W_{{\text {sp}}}^{(l)} (n_{{\text {e}}}-n_{{\text {tr}}}) n_{l}, \end{aligned}$$

2 $$\begin{aligned} \frac{dn_{l}}{dt}&= \left[ W_{{\text {sp}}}^{(l)} (n_{{\text {e}}} - n_{{\text {tr}}}) - \frac{1}{\tau _{{\text {p}}}}\right] n_{l}, \end{aligned}$$

where $\eta _{{\text {i}}}$ is an injection efficiency of the electrons into the active region, *I* is the injected current to the laser, $\tau _e$ is the electron recombination time, $n_{{\text {tr}}}$ is the number of electrons at transparency, and $\tau _{{\text {p}}}$ is the photon cavity lifetime for the lasing mode.

Here, the photon cavity lifetime accounts for the intrinsic losses in the cavity $\tau _{{\text {i}}}$ due to scattering and absorption, and losses due to useful output coupling $\tau _{{\text {ext}}}$. $W_{{\text {sp}}}^{(l)}$ is the spontaneous emission rate into the lasing mode (*l*), a material-dependent constant. We choose the total number of photons and the total number of excited electrons as our main variables rather than their densities since according to the quantum mechanics that describe the interaction between photons and electrons, a single electronic transition results in the emission or the absorption of one photon.

A key result of these equations is the intensity modulation (IM) response, defined as the ratio of the amplitude of the total emitted power modulation $\Delta P_{{\text {out}}}$ at a frequency *f* to the amplitude of the driving current $\Delta I$ at *f*, and is given by:

3 $$\begin{aligned} H(f)&= \frac{\Delta P_{{\text {out}}} (f) }{\Delta I (f)} = \frac{\eta _{{\text {d}}} h\nu /q}{1 - (f/f_{{\text {R}}})^2 + i(f/f_{{\text {R}}})( 2\pi f_{{\text {R}}} \tau _{{\text {p}}} + 1 /\left( 2 \pi f_{{\text {R}}} \tau _{{\text {e}}}\right) )}, \end{aligned}$$

4 $$\begin{aligned} f_{{\text {R}}}&= \frac{1}{2\pi }\sqrt{\eta _{{\text {i}}} \frac{W_{{\text {sp}}}^{(l)} (I - I_{{\text {th}}})}{q}}. \end{aligned}$$

This transfer function *H*(*f*) is that of a second-order low-pass filter response whose magnitude is constant at low frequencies. At higher frequencies, above that of the relaxation resonance frequency $f_{{\text {R}}}$, *H*(*f*) drops by 40 dB per decade. It is consequently used as a measure of the upper limit to the response of the laser intensity to current modulation. *H*(*f*), as will be shown below, also plays a key role in the laser temporal coherence. It can be seen from Eq. (4) that $f_R$ is proportional to the square root of the spontaneous emission rate determined by the strength of the interaction between the electrons and the lasing mode. Following an ab-initio analysis of the quantum interaction between the excited electron and the lasing mode, we find that the spontaneous and stimulated transition rates of an electron from an excited state in the conduction band to an unoccupied state in the valence band, due to the interaction with the lasing mode (*l*) in Eqs. (1) and (2), are given by^6,7,14^:

5 $$\begin{aligned} W_{{\text {sp}}}^{(l)}&= \frac{ 2\pi ^2 \mu ^2 \nu _l g_{{\text {a}}}\left( \nu _l \right) }{h\varepsilon \left( {\overline{r}}_{{\text {a}}}\right) } \left| {\overline{E}}_l\left( {\overline{r}}_a\right) \right| ^2, \end{aligned}$$

6 $$\begin{aligned} W_{{\text {st}}}^{(l)}&= n_l W_{{\text {sp}}}^{(l)}, \end{aligned}$$

where $\mu$ is the dipole transition matrix element, $g_{{\text {a}}}(\nu _l)$ is the value of the normalized lineshape function of the transition at the lasing frequency $\nu _l$ (both known quantities for our purposes). $\varepsilon \left( {\overline{r}}_a\right)$ is the permittivity of the bulk material at the location of the emitter. $\left| {\overline{E}}_l\left( {\overline{r}}_a\right) \right| ^2$ is the normalized intensity of the laser mode at the location of the emitter ${\overline{r}}_{{\text {a}}}$ (i.e., electron). Integrated over the volume of the quantum wells, $\left| {\overline{E}}_l(r_a)\right| ^2$ yields the confinement factor ($\Gamma$) of the electric field in the active region (i.e., $\left| {\overline{E}}_l(r_a)\right| ^2 \propto {\Gamma _{{\text {QW}}}}$)^15^. Combined with Eq. (4), we find that

7 $$\begin{aligned} f_{{\text {R}}} =\frac{1}{2\pi }\sqrt{\eta _{{\text {i}}} \frac{ 2\pi ^2 \mu ^2 \nu _l g_{{\text {a}}}\left( \nu _l \right) }{qh\varepsilon \left( {\overline{r}}_{{\text {a}}}\right) } \left| {\overline{E}}_l\left( {\overline{r}}_a\right) \right| ^2 \left( I - I_{{\text {th}}}\right) } \implies f_{{\text {R}}} \propto \left| {\overline{E}}_l\left( {\overline{r}}_{{\text {a}}}\right) \right| \sqrt{I - I_{{\text {th}}}}. \end{aligned}$$

This result highlights one key parameter in the control of the relaxation resonance frequency $f_R$: *the magnitude of the electric field at the location of the emitter*. Similarly, we obtain the damping coefficient $\gamma$ of the second-order response,

8 $$\begin{aligned} \gamma = \frac{1}{2}\left( 2\pi f_R\tau _p + \frac{1}{2\pi f_R \tau _e}\right) \propto \frac{1}{\left| {\overline{E}}_l\left( {\overline{r}}_{{\text {a}}}\right) \right| }. \end{aligned}$$

The results above are obtained using results from Eq. (7) and combining them with Equation 23 of^7^ which show that for the geometry of interest, $\tau _p \propto \left| {\overline{E}}_l\left( {\overline{r}}_{{\text {a}}}\right) \right| ^{-2}$. As a consequence, as we decrease $\left| {\overline{E}}_l\left( {\overline{r}}_{{\text {a}}}\right) \right|$, we find that the damping coefficient of the response increases giving these lasers an over-damped response to spontaneous emission generated in the gain region ensuring that the intensity noise is monotonically decreasing without the presence of any peak near the relaxation resonance frequency, which is observed in the case of an under-damped response.

**Figure 1:**
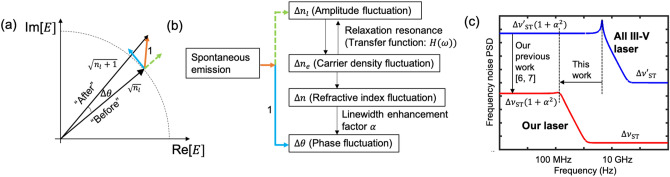
(**a**) The phasor model for the laser field phase, showing the electric field before and after a single spontaneous emission event (orange arrow). The projection of the spontaneous emission event onto the phase and intensity quadrature is shown in the blue solid and green dotted line respectively. (**b**) Phase noise due to spontaneous emission including both the direct spontaneous emission phase noise and the additional phase noise via the amplitude-phase coupling. (**c**) A cartoon illustrating the changes to the frequency noise power spectral density of the high-coherence Silicon/III–V laser studied as part of this work and the conventional III–V laser. In^6^ and^7^ we studied how the magnitude of the frequency noise power spectral density changes below the relaxation resonance frequency. In this work, we study how the location of the relaxation resonance frequency (Eq. 7), and the damping of this resonance (Eq. 8) are both reduced when we confine the majority of the optical mode within the low-loss silicon medium as opposed to the lossy but gain-providing III–V.

To understand how the relaxation resonance frequency affects the phase noise, we now look into the phase noise caused by the spontaneous emission events. At frequencies high enough where thermal noise can be ignored in semiconductor lasers, the spontaneous emissions become the dominant source of noise that perturbs the lasing field circulating in the cavity^10^. Furthermore, while thermal and electrical noise sources can be mitigated by improving the thermal and electronic design of the driving circuit, the origin of spontaneous emission is quantum mechanical and cannot be avoided thus determining the ultimate noise floor of the laser. These quantum perturbations can be analyzed as two distinct noise sources, one coupled to the amplitude quadrature, and the other coupled to the phase quadrature, as shown in Fig. 1a. The first component is perpendicular to the phasor of the electric field and couples directly to the phase of the optical field (blue solid line in Fig. 1a). The second component, parallel to the phasor of the field, alters the intensity of the lasing field (green dotted line in Fig. 1a). To compensate for the change in intensity, the gain-providing carriers fluctuate in response to these amplitude changes in an attempt to restore the steady-state output intensity of the laser (known as the “relaxation resonance”). In semiconductor quantum-well lasers, changes in the carrier concentration induce a corresponding proportional change in the refractive index leading to additional frequency noise through Kramers–Kroning relations^13^ due the asymmetric shape of the gain spectrum in this medium^16^. Thus, fluctuations in the total number of carriers manifest themselves as additional phase fluctuations through a process known as amplitude-phase coupling depicted in Fig. 1b.

To find a mathematical relationship between the quantum phase fluctuations of a laser and carrier dynamics, one can introduce Langevin noise terms in Eqs. (1) and (2), as is done in^11,17^. Doing so reveals that the amplitude-phase coupling has a frequency dependence identical to that of the intensity modulation response *H*(*f*), a second-order low pass filter response with a resonance frequency of $f_R$. Therefore, in the quantum-limit, the frequency noise PSD (single-sided) of a semiconductor laser can be expressed as the sum of two terms^14^,

9 $$\begin{aligned} S_{\Delta {\nu }}(f) = \frac{n_{{\text {2th}}} W_{{\text {sp}}}^{(l)}}{4 \pi ^2 n_{l}} (1 + \alpha ^2 \left| H(f)\right| ^2), \end{aligned}$$

where $n_{{\text {2th}}}$ is the total number of electrons in the conduction band at threshold. The first term $\frac{n_{{\text {2th}}} W_{{\text {sp}}}^{(l)}}{4\pi ^2 n_{l}}$is often referred to as the Schawlow–Townes linewidth, representing the intrinsic, quantum-limited, ultimate noise floor including only the direct spontaneous emission phase noise. The additional phase noise due to the amplitude-phase coupling appears with a coefficient of $\alpha ^2$ (a linewidth enhancement factor or the Henry $\alpha$ parameter), which ranges between 2 and 6 for the case of broadly-used quantum-well (QW) semiconductor lasers^15,18,19^, and it is modulated by $\left| H(f)\right| ^2$.

As such, for lasers with a relaxation resonance frequency $f_R\approx 10\,\hbox {GHz}$, the amplitude-phase coupling constitutes the majority of the spontaneous-emission-induced frequency noise for semiconductor lasers in the frequency bands of interest for high-speed sensing and communication with sampling rates in the few GHz, as shown in Fig. 1c. However, if one can design lasers with a relaxation resonance frequency positioned at the range of hundreds of MHz, then the total frequency noise of the lasers will drop by a factor of $(1+\alpha ^2)$ at frequencies of GHz leaving only the white noise floor generated by the direct spontaneous emission events.

In the rest of the paper, we will refer to the “enhanced linewidth” as the value that includes the carrier-induced frequency noise ($\Delta \nu _{{\text {enhanced}}} = \pi S_{\Delta \nu } (f < f_{{\text {R}}}) = \frac{n_{{\text {2th}}} W_{{\text {sp}}}^{(l)}}{4\pi n_{l}}(1 + \alpha ^2) = \Delta \nu _{{\text {ST}}}( 1+ \alpha ^2)$) and to the “Schawlow–Townes linewidth” as the value proportional to the direct contribution of spontaneous emission to phase noise ($\Delta \nu _{{\text {ST}}} = \pi S_{\Delta \nu }(f > f_{{\text {R}}})=\frac{n_{{\text {2th}}} W_{{\text {sp}}}^{(l)}}{4\pi n_{l}}$)^20^.

### Frequency noise above the relaxation resonance frequency of the high-coherence Silicon/III–V lasers

**Figure 2:**
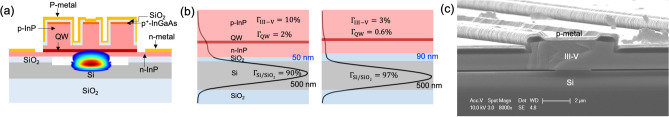
(**a**) Cartoon cross-sectional view of the laser device structure. (**b**) Transverse optical mode profile in the lasers with the 50 nm and 90 nm ${{\text {SiO}}}_2$ quantum noise control layer together with the confinement factor in each layer. **c** SEM image of the cross-section of the fabricated laser.

In our recently reported quantum noise controlled Silicon/III–V lasers^6,7^, we harnessed the Purcell effect to decrease the spontaneous emission rate into the lasing mode. This was achieved by utilizing a ${{\text {SiO}}}_2$ layer between the Silicon and III–V ranging from 50 nm to 150 nm, which we call the quantum noise control layer (QNCL), to engineer the optical mode’s spatial distribution with respect to the emitter ($\left| {\overline{E}}_l\left( {\overline{r}}_{{\text {a}}}\right) \right|$). The geometry of the cross-section and the transverse optical mode profile for the case of the 50 nm and 90 nm ${{\text {SiO}}}_2$ QNCL lasers are shown in Fig. 2b. Using this strategy, we have shown that Silicon/III–V lasers employing a monolithically integrated high-Q resonator can have linewidths as small as a few kHz without sacrificing the parameters such as the threshold current (due to the constant $n_{{\text {2th}}}$) and the optical output power^6,7^. The strategy is effective as long as the laser mode losses are dominated by absorption in the III–V or, equivalently, as long as the overall Q of the laser mode is dominated by III–V losses, which we estimate occurs when the thickness of the ${{\text {SiO}}}_2$ layer is approximately 150 nm. Further reduction in the electric field strength at the quantum-well layer results in the increase in the threshold carrier density to maintain the gain that matches the no-longer-decreasing modal loss, increasing the threshold current. In the batch of lasers fabricated as part of this study, the run on which the 150 nm lasers were fabricated failed. We are therefore focusing on discussion on the successfully fabricated 50 nm and 90 nm lasers.

To investigate the effect of the reduced electric field strength at the location of the quantum-wells on the relaxation resonance of the lasers, we revisit Eq. (7). Due to the dependence of the relaxation resonance frequency on the square root of the intensity of the electric field at the quantum-well emitter, every two orders of magnitude reduction in the field strength at the quantum-well will result in the reduction of one order of magnitude in the relaxation resonance frequency. In our demonstration, we’ve reduced $\Gamma _{{\text {III--V}}} = 100\%$ for conventional all III–V laser to $\Gamma _{{\text {III--V}}}=1\%$ ($\Gamma _{{\text {QW}}}=0.2\%$) for the case of the 150 nm QNCL laser resulting nearly in a full order of magnitude reduction in the relaxation resonance frequency of the laser from a few GHz to hundreds of MHz^21,22^.

Figure 1c illustrates that at frequencies of a few GHz, conventional III–V lasers still exhibit “enhanced frequency noise” including both the direct spontaneous emission phase noise and the amplitude-phase-coupling-induced frequency noise. In contrast, at a few GHz, our high-coherence Silicon/III–V lasers eliminate the additional frequency noise via amplitude-phase coupling by positioning a relaxation resonance frequency at a frequency of a few hundreds of MHz, yielding lasers that possess the intrinsic, quantum-limited Schawlow–Townes noise floor.

## Results and discussion

### Laser design and fabrication

The high-coherence Silicon/III–V laser is based on a Silicon waveguide to which a III–V epitaxially grown stack is bonded separated by a thin ${{\text {SiO}}}_2$ layer (Fig. 2a). The oxide layer is obtained by thinning a thermally grown ${{\text {SiO}}}_2$ layer, originally 400 nm thick, on the top of the silicon-on-insulator (SOI) waveguide using a buffer HF wet etch. The high-Q Silicon resonator is defined by etching a 60 nm rib on a 500 nm thick Silicon device layer creating a $2.5\,\upmu \hbox {m}$ wide waveguide. In the same fabrication steps, a 1-D grating is etched in the waveguide to define the optical cavity by etching holes 60 nm wide in the direction of propagation and of varying width.

The width of the gratings in the defection section varies between 200 nm and 300 nm in the transverse direction along the length of the resonator such that the $240\,\upmu \hbox {m}$-long defect has a photonic well that contains a single high-Q mode^6^. The intrinsic Q-factor of the fabricated Silicon resonator is measured to be as high as $10^6$. End reflections are provided by two mirror regions, on either side of the defect section, of $300\,\upmu \hbox {m}$ and $400\,\upmu \hbox {m}$ long for the 50 nm and 90 nm QNCL lasers, respectively. The mirror length of the 90 nm QNCL laser is chosen to be larger than that of the 50 nm QNCL laser to increase the loaded Q-factor of the 90 nm QNCL proportionally to the expected increase in the intrinsic Q-factor. The period of the grating determines the lasing wavelength, and in our case is chosen to be 240.0 nm and 237.5 nm for the 50 nm and 90 nm QNCL lasers, respectively. The unpatterned InP is directly bonded on the pattered SOI resonators and incorporates five InGaAsP quantum-wells. Subsequently, the mesa structure and the metal contacts are patterned on the III–V wafer^23^. Figure 2c shows the SEM image of the fabricated laser.

The mode has an estimated intrinsic Q-factor equal to the weighted harmonic mean of the Q-factors of the Silicon and III–V waveguides. Through finite element simulations, we estimate the confinement factor in the III–V of the 50 nm and 90 nm QNCL lasers to be 10% and 3% (Fig. 2b), respectively, yielding a Q-factor of approximately $1 \times 10^5$ and $2.5 \times 10^5$.

**Figure 3:**
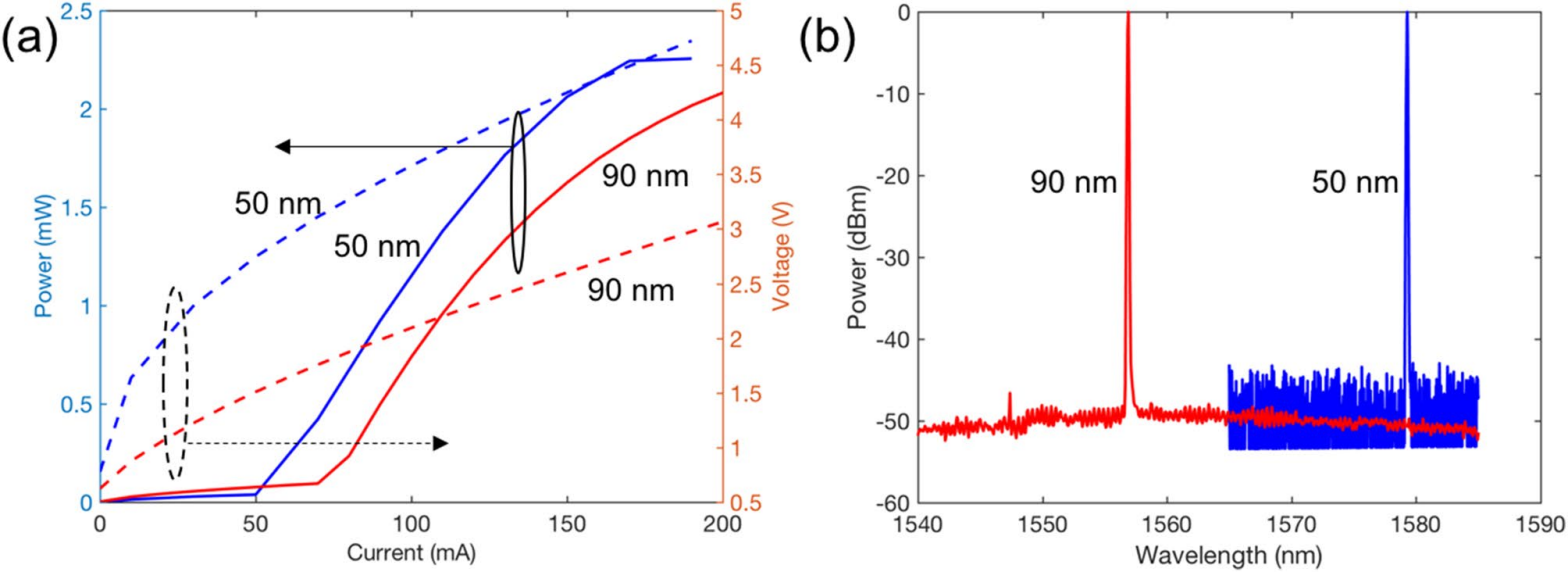
(**a**) The light vs. pump current (LI) and current vs. voltage (IV) characteristics of the 50 nm and 90 nm QNCL lasers at $20\,^{\circ }$C. (**b**) The optical spectrum of the lasers at $20\,^{\circ }$C, showing a single-mode operation with a side-mode suppression ratio larger than 45 dB.

### Measurements

The light-pump current (LI) and the current versus voltage (IV) characteristics of the fabricated 50 nm and 90 nm QNCL lasers are shown in Fig. 3a. The 50 nm and 90 nm laser obtain continuous-wave laser operation with threshold currents of 50 mA and 80 mA, respectively, and single-facet output powers more than 2 mW at $20\,^{\circ }\hbox {C}$. As theoretically predicted in^7^, the output power of both lasers is rather similar. This is due to the proportional decrease in both the gain and the losses within the laser cavity. We attribute the small variation in the laser output power to fabrication imperfections, often seen in small volume laser manufacturing processes. Figure 3b presents the optical spectrum of the 50 nm and 90 nm lasers obtaining a single-mode operation with side-mode suppression ratios greater than 45 dB at the lasing wavelength of 1577 nm and 1556 nm, respectively.

**Figure 4:**
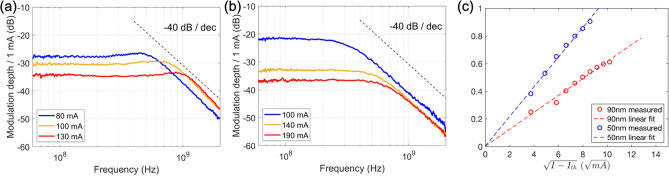
(**a**) The intensity modulation response of the 50 nm QNCL laser at different bias currents. (**b**) The intensity modulation response of the 90 nm QNCL laser at different bias currents. Measured using an HP8722C (50 MHz–40 GHz) and Optilab BPR-20-M (Up to 20 GHz). (**c**) Relaxation resonance frequencies of the 50 nm and 90 nm QNCL lasers as a function of $\sqrt{I-I_{{\text {th}}}}$.

The intensity modulation (IM) response *H*(*f*) of the lasers is measured using a network analyzer (HP 8722C) and a high-speed photodetector (Optilab BPR-20-M). The IM index *m*, defined as $\Delta P / P_0$ per 1 mA where $\Delta P$ is the change in the optical power and $P_0$ is the average received optical power, is shown in Fig. 4a for the 50 nm QNCL laser at bias currents of 80, 100, and 130 mA. As expected, the IM response exhibits a second-order low-pass filter behavior with flat response for frequencies up to the relaxation resonance frequencies, and 40 dB/decade drop-off thereafter. Fitting the measured response to the second-order low-pass filter response in Eq. (3) yields a relaxation resonance frequencies $f_{{\text {R}}}$ of 500, 730, and 900 MHz at bias currents of 80, 100, and 130 mA, respectively. The relaxation resonance frequencies of the 90 nm QNCL laser, extracted from the data presented in Fig. 4b, are measured to be 360, 540, and 680 MHz at bias currents of 100, 140, and 190 mA, respectively. These low relaxation resonance frequencies at hundreds of MHz clearly stand in contrast to those of conventional III–V lasers of a few GHz. The linear dependence of the relaxation resonance frequencies of the 50 nm and 90 nm QNCL lasers on $\sqrt{I - I_{{\text {th}}}}$, described in Eq. (7) is shown in Fig. 4c. The reduction in the relaxation resonance frequencies $f_{{\text {R}}}$ is observed with the increasing QNCL thickness, which is attributed to the reduced electric field strength $\left| {\overline{E}}_l\left( {\overline{r}}_{{\text {a}}}\right) \right|$ at the location of the quantum-wells (i.e., reduced $\Gamma _{{\text {QW}}}$). It can also be observed that the relaxation resonance peak is less apparent on the 90 nm QNCL laser compared to that of the 50 nm QNCL, due to the increased damping in the 90 nm QNCL as predicted in Eq. (8).

For the fabricated lasers, we found that the 50 nm QNCL laser, which has a lasing wavelength of 1577 nm, shows a linewidth enhancement factor of 5.8, whereas the 90 nm laser lasing at the wavelength of 1556 nm has a linewidth enhancement factor of 3. The details of the measurements follow the measurement setup and analysis of^15,24,25^ and are discussed in the Supplementary Material.

To finally characterize the linewidth of the lasers, we measure the frequency noise PSD using an RF spectrum analyzer after mixing the laser light with the time-delayed version of itself at the output of the 1.575 GHz MZI. A low frequency (100 Hz) feedback loop uses a piezo fiber stretcher to keep the quadrature point of the MZI locked to the wavelength of the laser for the duration of the measurement. The measured PSD of the photocurrent fluctuation is converted to the frequency noise PSD of the laser^6^.

**Figure 5:**
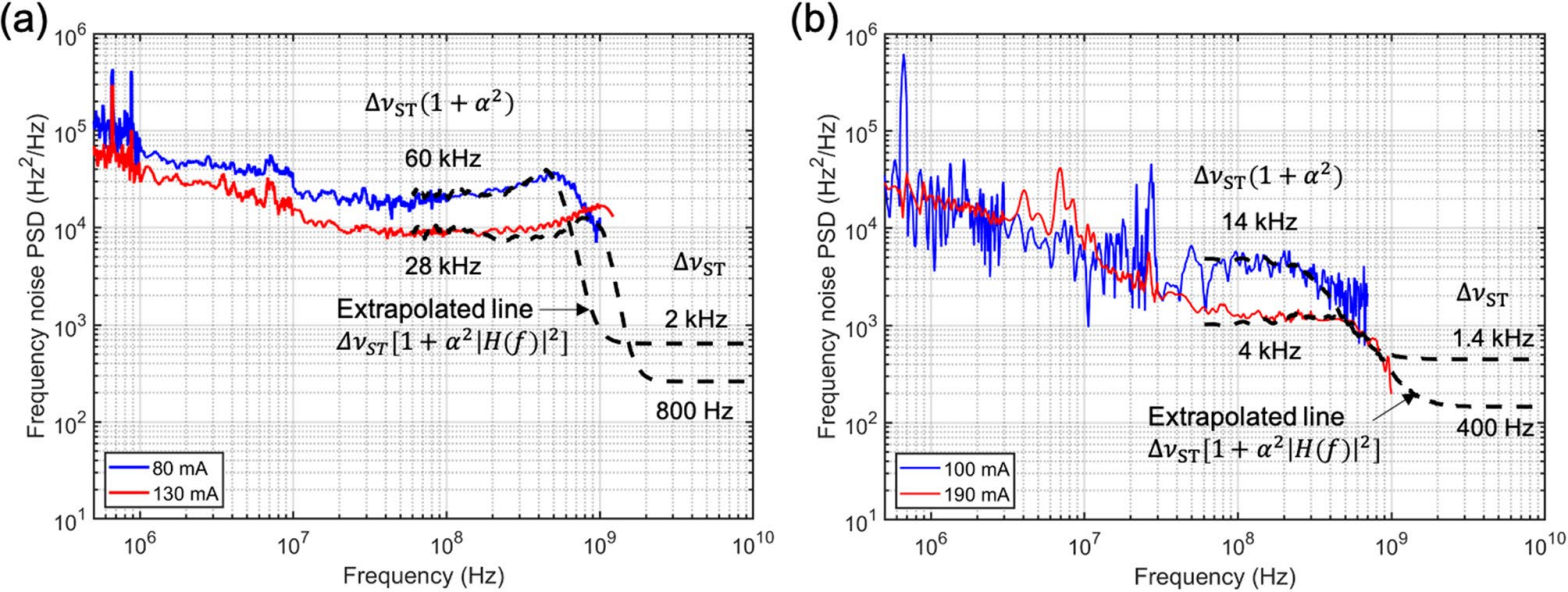
The frequency noise power spectral density of the lasers. The expected frequency noise at frequencies further into the GHz range is extrapolated in black dotted line using Eq. (9). (**a**) The 50 nm QNCL laser ($I_{{\text {th}}} = 50\,\hbox {mA}$, $\lambda _0 = 1577\,\hbox {nm}$, $\alpha = 5.8$). (**b**) The 90 nm QNCL laser ($I_{{\text {th}}} = 80\,\hbox {mA}$, $\lambda _0 = 1556\,\hbox {nm}$, $\alpha = 3.0$).

The frequency noise PSD of the 50 nm QNCL laser ($I_{{\text {th}}} = 50\,\hbox {mA}$, $\lambda _0 = 1577\,\hbox {nm}$) is shown in Fig. 5a. The steep 1/*f* noise is observed in the low frequency region up to 10 MHz. The enhanced linewidth can be estimated when the spectrum reaches a flat white frequency noise floor between frequencies of 10 and 100 MHz, where thermal noise is heavily suppressed. In this range, the PSD ($S_{\Delta \nu }$) takes a value of $1.9 \times 10^4\,{{\text {Hz}}}^2/{{\text {Hz}}}$ for a bias current of 80 mA corresponding to an enhanced linewidth of 60 kHz. When the current is increased to 130 mA, the frequency noise drops to $8.9 \times 10^3\,{{\text {Hz}}}^2/{{\text {Hz}}}$, corresponding to an enhanced linewidth of 28 kHz.

To characterize the Schawlow–Townes linewidth observable at frequencies above the relaxation resonance frequency $f_{{\text {R}}}$, a direct measurement of the spontaneous emission white noise floor is desirable. However, the finite output power of the lasers in this proof-of-concept demonstration made their frequency noise approach the shot-noise level at approximately 1 GHz just above the relaxation resonance frequency. As a consequence, it was not possible to measure the frequency noise much above the relaxation resonance frequency. However, we are able to observe the early effects of the low relaxation resonance frequency and its peak in the frequency range between 100 MHz and 1 GHz, which shows excellent agreement with the intensity modulation response measured in Fig. 4a. Using Eq. (9), we can extract an estimate of the Schawlow–Townes linewidth at frequencies above $f_{{\text {R}}}$ by combining the measured PSD $\Delta \nu _{{\text {enhanced}}} = \Delta \nu _{{\text {ST}}} (1 + \alpha ^2)$ of the lasers below $f_{{\text {R}}}$, measurements of the modulation response *H*(*f*) and linewidth enhancement factor $\alpha \approx 5.8$. We can see that the frequency noise estimate (the dashed line in Fig. 5a) extrapolated based on $\Delta \nu _{{\text {enhanced}}}$, *H*(*f*), $\alpha$ and the measured curves at both currents are in good agreement up to 1 GHz. Therefore, from the extrapolated estimate, we predict that the frequency noise above $f_{{\text {R}}}$ at a bias current of 80 mA is $540\,{{\text {Hz}}}^2/{{\text {Hz}}}$, yielding a Schawlow–Townes linewidth of 1.7 kHz. For the bias current of 130 mA, the frequency noise above $f_{{\text {R}}}$ is estimated to be $250\,{{\text {Hz}}}^2/{{\text {Hz}}}$, corresponding to Schawlow–Townes linewidth of 0.8 kHz, achieving yet another order of magnitude decrease in the frequency noise.

The early effects of the low relaxation resonance frequency of the 90 nm QNCL laser ($I_{{\text {th}}} = 80\,\hbox {mA}$, $\lambda _0 = 1556\,\hbox {nm}$) are also observable in Fig. 5b where it can be seen that the frequency noise power spectral density decreases near the relaxation resonance frequency—360 MHz and 680 MHz, as measured in the IM response, for pump currents of 100 mA and 190 mA respectively. The frequency noise estimate for the 90 nm laser is extrapolated using $\Delta \nu _{{\text {enhanced}}}$, *H*(*f*), $\alpha$ of this laser and is shown in the dashed line in Fig. 5b. The measured and the extrapolated lines show good agreement with each other up to approximately 1 GHz. Hence, we measure a frequency noise floor above $f_{{\text {R}}}$ to be $450\,{{\text {Hz}}}^2/{{\text {Hz}}}$ at a bias current of 100 mA, yielding a Schawlow–Townes linewidth of 1.4 kHz. At a bias current of 190 mA, the frequency noise above $f_{{\text {R}}}$ is expected to be $130\,{{\text {Hz}}}^2/{{\text {Hz}}}$, achieving the Schawlow–Townes linewidth of 0.4 kHz.

### Discussion

In this paper, we showed that the strategy of quantum noise control, i.e., control of the spontaneous emission rate, can decrease the relaxation resonance frequency $f_{{\text {R}}}$ of semiconductor lasers to a few hundred MHz, resulting in the suppression of the phase noise induced by the amplitude-phase coupling and reduction in the frequency noise PSD by an additional order of magnitude. Hence, we theoretically and experimentally demonstrated that in order to decrease the frequency noise of semiconductor lasers, all three “knobs” in Eq. (9), the spontaneous emission rate $W_{{\text {sp}}}^{(l)}$, the number of the stored photons $n_l$, and the transfer function of the modulation response *H*(*f*) can be controlled and optimized by the low loss modal design which stores the great majority of the optical modal energy in a low-loss material rather than in the active, high-loss material.

In this work, the concept of reducing the laser phase noise by decreasing the modal loss is realized through modal engineering in the transverse direction of the waveguide. The concept of increasing the Q-factor of the laser cavity through the modal engineering is also possible in the longitudinal direction through the use of tapers to transition the optical mode between the III–V and passive low-loss waveguide, as the wave travels along the length of the resonator^26,27^. In these architectures, the confinement factor in the active region scales as the ratio of the length of the active region to the effective length of the cavity. This means that reducing the relaxation resonance frequency by an order of magnitude would require the size of the external cavity laser to scale by two orders of magnitude. However, in the lasers described here, small changes in the thickness of the ${{\text {SiO}}}_2$ layer on the order of tens of nanometers can change the confinement factor by a factor of 100 and can alter the relaxation resonance frequency of the laser by an order of magnitude. The transverse modal control allows us to achieve the same effect with very little compromise in footprint. Furthermore, keeping a small footprint for the laser helps ensure mode-hop free operation even as the injection current is changed.

While the amplitude-phase coupling term that originates from the gain medium in the laser causes linewidth broadening, it is important to note that other dispersive medium, whether in the form of a reflector or a mild absorber, can be introduced in the laser cavity to decrease the laser linewidth^28,29^. This is equivalent to the introduction of a negative feedback for the phase of the laser. While these techniques are not directly explored as part of this paper, they typically introduce the reflector outside the main laser cavity and as such, are complimentary to the result discussed below.

It is notable that the magnitude of the linewidth enhancement factor $\alpha$ is an intrinsic property of the *gain medium*, regardless of the structure of the optical mode in the laser. Therefore, for a given material, one cannot eliminate the presence of the linewidth enhancement factor and its effect on the phase noise. Other methods used to suppress the linewidth enhancement factor include the use of quantum-dots (QDs) as the gain medium. Quantum-dots do not exhibit a linewidth enhancement factor, due to their delta function-like density of states^30,31^. However, quantum-dots still remain difficult to grow for certain materials and often exhibit lower material gain than their quantum-well counterparts. The demonstration of the suppression of the linewidth enhancement factors at a relatively low frequency in our lasers means that using this approach, lasers can obtain an effective linewidth enhancement factor of 0 in the GHz range without utilizing quantum dots as the gain providing material.

Of further importance is the dependence of the linewidth enhancement factor on the operating wavelength of the laser. As discussed in^24,25^ and in agreement with our measurements of $\alpha$ for lasers of different operating wavelengths, lasers that operate further from the differential gain peak experience a larger linewidth enhancement factor. Lasers designed with the same gain medium operating at different wavelengths will consequently have different noise characteristics, owing to differences in the linewidth enhancement factor. This is of special importance for tunable laser designs or dense laser arrays^32^. By having low relaxation resonance frequencies, the influence of the linewidth enhancement factor can be suppressed ensuring that lasers operating at different wavelengths will have nearly identical spectral characteristics at a few GHz range.

## Conclusion

In previous work, we have shown that our QNCL laser design leads to in addition to orders of magnitude reduction of phase noise and to isolator-free operation. Here, we have demonstrated theoretically and experimentally that our low-loss laser modal design leads to an important third consequence: that relaxation resonance frequencies can be made as low as a few hundreds of MHz for compact heterogeneously integrated Silicon/III–V lasers. These low relaxation resonance frequencies enable the lasers to achieve the quantum-limited Schawlow–Townes linewidth, as low as 0.4 kHz, by effectively reducing to insignificance the amplitude-phase coupling (characterized by the Henry $\alpha$ parameter). As new fabrication techniques continue to be refined and new optical materials emerge, this vertical optical modal engineering strategy without requiring any sort of external cavity can be useful for creating compact, even lower noise lasers that do not suffer from additional phase noise due to amplitude-phase coupling at a variety of operating wavelengths.

## Supplementary Information

Supplementary Information.
