# Supplementary material for: Consequences of quantum noise control for the relaxation resonance frequency and phase noise in heterogeneous Silicon/III–V lasers
Source: Sci Rep. 2022 Jan 10;12:312. doi: 10.1038/s41598-021-03314-8 (PMC8748436; doi:10.1038/s41598-021-03314-8)
Supplement: Supplementary file 1 — Supplementary Information. [file 41598_2021_3314_MOESM1_ESM.pdf]

Consequences of quantum noise control for the relaxation resonance frequency and phase noise in heterogeneous Silicon/III-V lasers - Supplementary Material

Dongwan Kim[1,*,+], Mark Harfouche[2,+], Huolei Wang[1,†], Christos T. Santis[1], Yaakov Vilenchik[1], Naresh Satyan[3], George Rakuljic[3], and Amnon Yariv[1,2]

[1]Department of Applied Physics and Materials Science, California Institute of Technology, Pasadena, California 91125, USA
[2]Department of Electrical Engineering, California Institute of Technology, Pasadena, California 91125, USA
[3]Telaris Inc., Santa Monica, California 90403, USA
[*]Corresponding author: dongwan.kim@caltech.edu
[+]these authors contributed equally to this work

Measurement of the linewidth enhancement factor

The ratio between the FM index M and IM index m can be used to extract the linewidth enhancement factor of the laser using the relation $\alpha = M/(m/2)$[1]. The FM index M is defined as $\Delta f/f_{\text{mod}}$, where Δf is the change in the optical frequency and f_{mod} is the modulation frequency. The FM response can be measured using the same network analyzer and high-speed photodetector as in the IM response. A Mach-Zehnder interferometer (MZI) with a free spectral range (FSR) of 1.575 GHz serves as a frequency discriminator by converting phase modulation into intensity modulation. Figure S1 (a) shows the measured FM index M, of the 50 nm QNCL laser together with the IM index m at a bias current of 130 mA. It is clear that both the IM and FM response exhibit the expected second-order low-pass filter response[2]. The FM response also shows the peak at the same relaxation resonance frequency of 900 MHz, as does the IM response. At low modulation frequencies, the FM response exhibits an additional modulation contribution from the thermo-optic effect. The linewidth enhancement factor α, obtained from the ratio between the FM and IM index, of the 50 nm QNCL laser is shown in Figure S1 (b). At frequencies above 1 GHz, the thermal response is heavily suppressed, and only the carrier modulation effect remains. Here, the ratio $M/(m/2)$ converges to a value of the linewidth enhancement factor $\alpha \approx 5.8$, regardless of the bias current level. The measured 90 nm QNCL laser, performed in a similar manner, yields a value of $\alpha \approx 3$. We observe that the 50 nm QNCL laser, which has a lasing wavelength

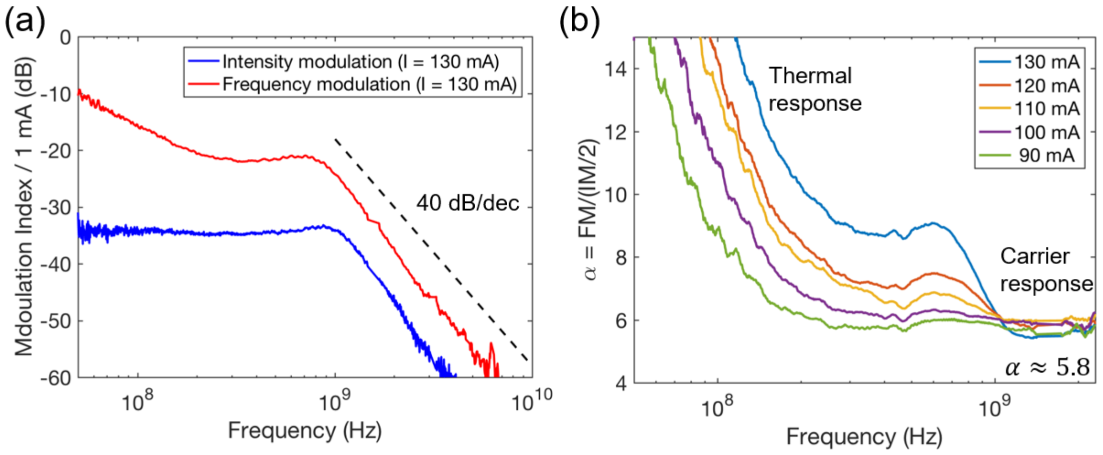

Figure S1. (a) The intensity and frequency modulation response of the 50 nm QNCL laser at the bias current of 130 mA. (b) The calculation of the linewidth enhancement factor of the 50 nm QNCL laser.

of 1577 nm, shows a linewidth enhancement factor of 5.8, whereas the 90 nm laser lasing at the wavelength of 1556 nm has a linewidth enhancement factor of 3. As discussed in[1,3], an increase in the linewidth enhancement factor is observed, as the lasing wavelength moves to higher frequencies compared to the differential gain peak.

References

1. Vahala, K., Chiu, L. C., Margalit, S. & Yariv, A. On the linewidth enhancement factor α in semiconductor injection lasers. *Appl. Phys. Lett.* **42**, 631–633, DOI: 10.1063/1.94054 (1983). http://dx.doi.org/10.1063/1.94054.
2. Coldren, L. A., Corzine, S. W. & Mashanovitch, M. L. *Diode Lasers and Photonic Integrated Circuits* (Wiley, 2012), 2nd ed edn.
3. Zhao, B. *et al.* Direct measurement of linewidth enhancement factors in quantum well lasers of different quantum well barrier heights. *Appl. Phys. Lett.* **62**, 1591–1593, DOI: 10.1063/1.108647 (1993). http://dx.doi.org/10.1063/1.108647.
